# The Effectiveness of Plecanatide for Treating Constipation and Bloating in Patients Aged 18 to 40 Years With Irritable Bowel Syndrome: Utilization of a New Composite Trisymptom Endpoint

**DOI:** 10.1111/jgh.70119

**Published:** 2025-11-10

**Authors:** Darren M. Brenner, Andrea S. Shin, Adam P. Laitman, Jill K. Deutsch, David C. Kunkel

**Affiliations:** ^1^ Internal Medicine‐Gastroenterology Northwestern University–Feinberg School of Medicine Chicago Illinois USA; ^2^ Vatche and Tamar Manoukian Division of Digestive Diseases, Department of Medicine University of California, Los Angeles Los Angeles California USA; ^3^ Salix Pharmaceuticals Bridgewater New Jersey USA; ^4^ Section of Digestive Diseases, Department of Internal Medicine Yale School of Medicine New Haven Connecticut USA; ^5^ Division of Gastroenterology University of California San Diego La Jolla California USA

**Keywords:** abdominal pain, bloating, constipation, guanylate cyclase C agonists, irritable bowel syndrome

## Abstract

**Background and Aim:**

Irritable bowel syndrome with constipation (IBS‐C) is characterized by multiple sensory symptoms, including abdominal pain, bloating, and bowel habit alterations. Therapeutic response should address all components. This study assesses a new exploratory trisymptom composite efficacy endpoint in an IBS‐C population of young adults with bloating treated with plecanatide.

**Methods:**

Pooled data were analyzed from two phase 3, randomized, double‐blind trials. Patients (18–40 years) with IBS‐C and baseline bloating (score ≥ 1) received plecanatide 3 mg or placebo for 12 weeks. The composite response definition was simultaneous improvement from baseline in three symptoms (abdominal pain, bloating, and complete spontaneous bowel movements [CSBMs]/week) for ≥ 6 of 12 weeks using several thresholds (≥ 2‐point or ≥ 30% or ≥ 40% improvement in abdominal pain and bloating plus an increase of ≥ 1 or ≥ 2 CSBMs in the same week).

**Results:**

Six hundred and five adults were included (plecanatide [*n* = 313]; placebo [*n* = 292]). Plecanatide/placebo baseline mean symptom scores were 6.2/6.4 for abdominal pain and 6.4/6.6 for bloating; both had a mean of 0.2 CSBMs/week. Significantly more patients in plecanatide versus placebo groups (*p* ≤ 0.01 for all comparisons) were trisymptom composite responders by several stringent thresholds, including ≥ 30% improvement in pain and bloating plus ≥ 1 CSBM/week increase (23.3% vs. 13.4%; *p* = 0.002) and ≥ 30% improvement in pain and bloating plus ≥ 2 CSBMs/week increase (19.5% vs. 8.9%; *p* < 0.001). Plecanatide was well tolerated.

**Conclusion:**

Plecanatide simultaneously and significantly improved combined symptoms of abdominal pain, bloating, and CSBM frequency at varying thresholds. Plecanatide is effective in improving global IBS‐C symptoms in individuals with bloating.

**Trial Registration:** ClinicalTrials.gov identifiers—NCT02387359 and NCT02493452.

## Graphical abstract

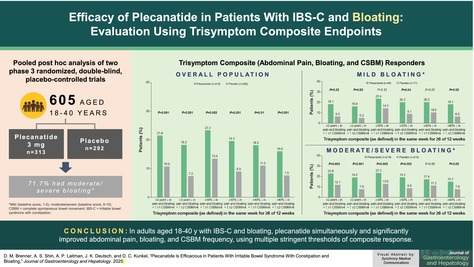

## 1 Introduction

Irritable bowel syndrome (IBS) is a heterogeneous disorder of gut‐brain interaction (DGBI) characterized as recurrent abdominal pain associated with defecation and/or a change in bowel habits, and there is a higher prevalence in adults < 50 years of age [1, 2, 3]. Patients with IBS also commonly have other sensory‐related symptoms in addition to abdominal pain, including bloating [1]. While bloating is one of the most common symptoms identified in patients who have IBS with constipation (IBS‐C) [4, 5, 6, 7], with most patients reporting moderate to severe intensity [5], it is not currently a criterion used to define IBS [1]. This is despite the fact that bloating prompts many individuals to seek medical care [8, 9] and is associated with poorer health‐related quality of life in patients with IBS‐C compared with those without bloating [10]. A survey of 467 individuals with IBS‐C reported that 28% of respondents “always” (scale, 1 [*never*] to 5 [*always*]) experienced bloating during the previous 3 months [5]. In addition, bloating is perceived as a potentially challenging symptom to treat [11]. Therefore, it is important to identify medications that effectively address and improve this symptom in addition to the cardinal symptoms of abdominal pain and altered bowel habits.

Plecanatide is a guanylate cyclase‐C agonist indicated for the treatment of chronic idiopathic constipation and IBS‐C in adults (approved dose, 3 mg once daily) [12]. Multiple phase 3, randomized, double‐blind, placebo‐controlled trials have demonstrated that plecanatide improves gastrointestinal symptoms, including bowel movement frequency [13, 14, 15]. Plecanatide has also been shown to improve IBS‐C symptoms in patients across varying levels of baseline bloating severity [16]. Given that bloating in adults is more likely to occur in those aged < 40 years compared with older age groups [17], including in those with IBS‐C [18], the aim of this study was to evaluate the efficacy of plecanatide in young adults with IBS‐C who had bloating at baseline. A new exploratory trisymptom composite endpoint was utilized to assess improvement in patients with classically defined (Rome criteria) IBS symptoms plus bloating at various thresholds of response.

## 2 Methods

Data were pooled and analyzed post hoc from two identically designed, phase 3, randomized, double‐blind, placebo‐controlled US trials that have been described previously (ClinicalTrials.gov identifiers NCT02387359 and NCT02493452) [13]. Participants included in the current analyses were males or females aged 18 to 40 years with IBS‐C (Rome III criteria), baseline bloating (score ≥ 1 on a scale of 0 [*no*] to 10 [*worst possible*]), and body mass index between 18 and 40 kg/m^2^. Baseline symptom scores were determined from electronic diary data collected during a 2‐week screening phase. Per the original protocols, inclusion criteria included a stable diet for ≥ 30 days before the first visit, with an expectation of stable dietary intake during the studies. Both trials received institutional review board approval at each center and were conducted in accordance with the ethical principles of the Declaration of Helsinki. All participants provided written informed consent prior to study participation.

Patients were stratified by sex and then randomly assigned (1:1:1) using an interactive web‐based response system to receive plecanatide 3 mg (approved dose), plecanatide 6 mg, or placebo once daily for 12 weeks [13]. The plecanatide 6‐mg arm was not included in the current analyses, as this dose is not approved for the treatment of IBS‐C due to a lack of additional treatment benefit compared with the 3‐mg dose [12]. Through 2‐week posttreatment, abdominal pain and bloating intensity were each separately assessed, using an 11‐point scale (range, 0 [*no*] to 10 [*worst possible*]), and frequency and completeness of bowel movements were recorded daily in an electronic diary. Response was defined as simultaneous improvement from baseline in all three symptoms—abdominal pain, bloating, and complete spontaneous bowel movements (CSBMs)—each week for ≥ 6 of the 12 weeks of treatment. Several composite criteria thresholds were evaluated, based on clinical experience and consistency with previously evaluated minimum thresholds for individual and two‐symptom composite endpoints in IBS‐C (i.e., ≥ 2‐point decrease or ≥ 30% or ≥ 40% improvement in abdominal pain and bloating plus an increase of ≥ 1 or ≥ 2 CSBMs in the same week for ≥ 6 of 12 weeks). Patients were also stratified by baseline bloating intensity (mild [score, 1–5]; moderate/severe [score, 6–10]). If a patient had 4 to < 7 compliant diary entries during a week, any missing entries were counted as zero bowel movements in the calculation of weekly frequency. For abdominal pain and bloating, the mean weekly score during a particular week for a patient was the average of nonmissing diary entries for each symptom. *p‐*values were calculated using the Cochran–Mantel–Haenszel test, with stratification by gender and a two‐sided significance level of 0.05.

## 3 Results

A total of 605 patients aged 18 to 40 years with IBS‐C and any intensity of bloating at baseline were included in the analysis (Table 1). Of these patients, 514 (85.0%) completed the study through Week 12, with the most common reasons for study discontinuation being withdrawal of consent (*n* = 36) or loss to follow‐up (*n* = 29). Most of the 605 patients were female (71.6%) and had moderate to severe bloating at baseline (71.7%). Demographic and baseline characteristics were comparable between the plecanatide (*n* = 313) and placebo groups (*n* = 292), including mean bloating score, abdominal pain score, and CSBMs/week (Table 1).

**TABLE 1:** Demographics and baseline characteristics.

Parameter	Plecanatide 3 mg (*n* = 313)	Placebo (*n* = 292)
Age, year		
Mean (SD)	30.6 (6.3)	30.3 (6.6)
Median (range)	31.0 (18–40)	31.0 (18–40)
Sex, *n* (%)		
Female	220 (70.3)	213 (72.9)
Male	93 (29.7)	79 (27.1)
BMI, kg/m^2^, mean (SD)	27.3 (4.8)^a^	27.0 (4.6)
Race/ethnicity, *n* (%)		
White	237 (75.7)	226 (77.4)
Black	59 (18.8)	54 (18.5)
Asian	13 (4.2)	8 (2.7)
Other	4 (1.3)	4 (1.4)
Abdominal pain score, mean (SD)^b^	6.2 (1.7)	6.4 (1.7)
Bloating score, mean (SD)^b^	6.4 (1.7)	6.6 (1.7)
Bloating intensity, *n* (%)		
Mild (score, 1–5)	94 (30.0)	77 (26.4)
Moderate/severe (score, 6–10)	219 (70.0)	215 (73.6)
CSBMs/week, mean (SD)	0.2 (0.5)	0.2 (0.5)

Abbreviations: BMI = body mass index; CSBM = complete spontaneous bowel movement.

^a^ Data missing for one patient.

^b^ Abdominal pain and bloating were separately measured using an 11‐point scale (range, 0 [*no*] to 10 [*worst possible*]).

**FIGURE 1:**
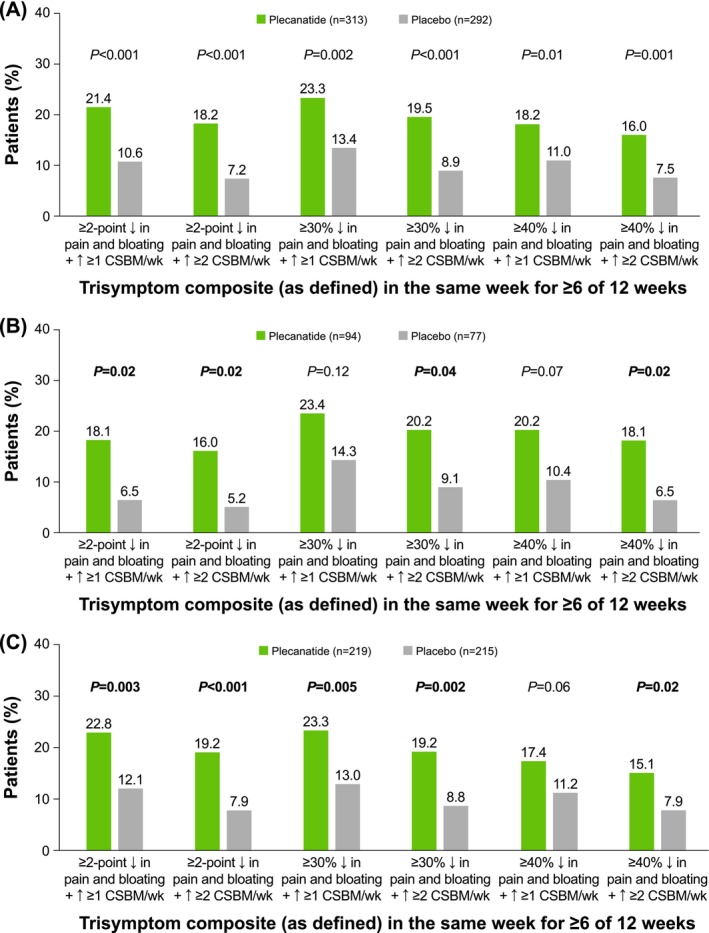
IBS‐C trisymptom composite responders in the (A) overall population and in patients with (B) mild or (C) moderate/severe bloating at baseline. IBS‐C = irritable bowel syndrome with constipation; CSBM = complete spontaneous bowel movement.

**FIGURE 2:**
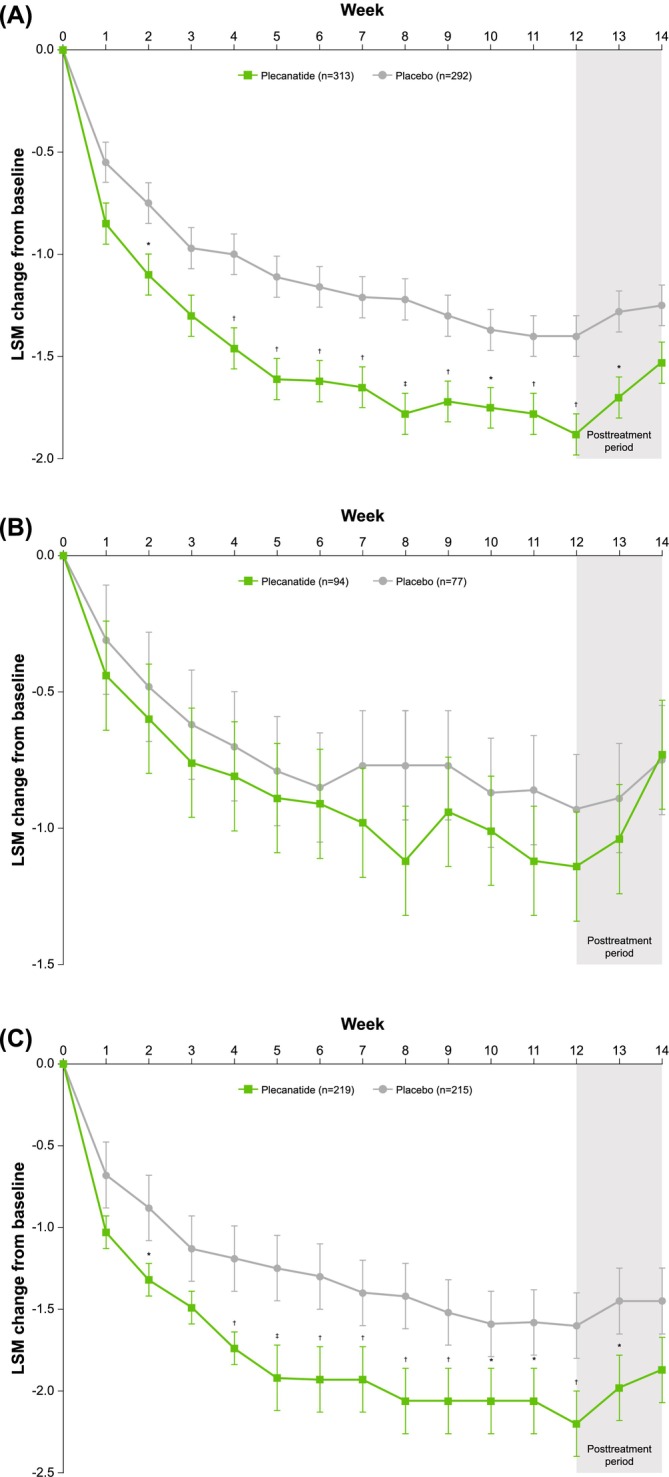
LSM change from baseline in bloating in the (A) overall population and in patients with (B) mild or (C) moderate/severe bloating at baseline. *p < 0.05. ^†^ *p* ≤ 0.01. ^‡^ *p* ≤ 0.001. LSM = least‐squares mean.

Overall, a significantly larger percentage of patients treated with plecanatide compared with placebo were trisymptom composite responders, defined using several stringent thresholds for response (*p* ≤ 0.01 vs. placebo for all comparisons; Figure 1A). The thresholds defining trisymptom response included a ≥ 2‐point or ≥ 30% or ≥ 40% simultaneous improvement from baseline in abdominal pain and bloating scores, plus an increase of either ≥ 1 or ≥ 2 CSBMs per week for ≥ 6 of the 12 weeks of treatment. An assessment of the least‐squares mean (LSM) change from baseline in bloating by week showed a statistically significant improvement with plecanatide compared with placebo during Weeks 2 through 12 (except Week 3; Figure 2A). After treatment discontinuation, improvement in bloating decreased as expected during the 2‐week follow‐up period (Figure 2A).

Additional analyses further stratified patients by bloating intensity (mild; moderate/severe). Patients in the mild subgroup had a mean baseline bloating score of 4.3 in both the plecanatide and placebo arms; in the moderate/severe subgroup, the mean baseline bloating score in both arms was 7.4 (Table 2). Similarly, the mean baseline abdominal pain score was approximately 4.4 for both the plecanatide and placebo arms in the mild bloating subgroup and 7.0 for both arms in the moderate/severe bloating subgroup. Significant improvements from baseline favoring plecanatide compared with placebo were noted for most of the stringent trisymptom composite endpoints analyzed (Figure 1B,C). For the mild bloating subgroup, a significantly larger percentage of responders was observed for plecanatide versus placebo for 4 of the 6 categories defining response (Figure 1B). Notably, the superiority of plecanatide versus placebo was observed for the most stringent definition (≥ 40% improvement in pain and bloating, plus an increase of ≥ 2 CSBMs/week in the same week for ≥ 6 of 12 treatment weeks; 18.1% vs. 6.5%, respectively; *p* = 0.02). An assessment of the LSM change from baseline for the individual symptom of bloating by week showed a numerical, but not statistically significant, difference between plecanatide and placebo in patients with mild bloating (Figure 2B).

**TABLE 2:** Demographic and baseline characteristics by bloating intensity.

Parameter	Mild bloating^a^		Moderate/severe bloating^b^	
	Plecanatide 3 mg (*n* = 94)	Placebo (*n* = 77)	Plecanatide 3 mg (*n* = 219)	Placebo (*n* = 215)
Age, year				
Mean (SD)	29.2 (6.6)	31.1 (6.3)	31.1 (6.1)	30.0 (6.7)
Median (range)	28.5 (18–40)	31.0 (18–40)	32.0 (18–40)	31.0 (18–40)
Sex, *n* (%)				
Female	65 (69.1)	51 (66.2)	155 (70.8)	162 (75.3)
Male	29 (30.9)	26 (33.8)	64 (29.2)	53 (24.7)
BMI, kg/m^2^, mean (SD)	26.3 (5.0)^c^	26.8 (4.4)	27.8 (4.6)	27.1 (4.7)
Race/ethnicity, *n* (%)				
White	62 (66.0)	59 (76.6)	175 (79.9)	167 (77.7)
Black	20 (21.3)	12 (15.6)	39 (17.8)	42 (19.5)
Asian	11 (11.7)	4 (5.2)	2 (0.9)	4 (1.9)
Other	1 (1.1)	2 (2.6)	3 (1.4)	2 (0.9)
Abdominal pain score, mean (SD)^d^	4.4 (0.7)	4.5 (0.8)	7.0 (1.3)	7.0 (1.4)
Bloating score, mean (SD)^d^	4.3 (0.8)	4.3 (0.9)	7.4 (1.1)	7.4 (1.1)
CSBMs/week, mean (SD)	0.2 (0.5)	0.4 (0.5)	0.2 (0.5)	0.2 (0.4)

Abbreviations: BMI = body mass index; CSBM = complete spontaneous bowel movement.

^a^ Score, 1–5.

^b^ Score, 6–10.

^c^ Data missing for one patient.

^d^ Abdominal pain and bloating were separately measured using an 11‐point scale (range, 0 [*no*] to 10 [*worst possible*]).

For the moderate/severe bloating subgroup, a significantly larger percentage of responders was observed for plecanatide versus placebo for 5 of the 6 categories defining response (Figure 1C). This included a significant difference favoring plecanatide versus placebo for the most stringent definition (≥ 40% improvement in pain and bloating plus an increase of ≥ 2 CSBMs/week in the same week for ≥ 6 of 12 treatment weeks; 15.1% vs. 7.9%; *p* = 0.02). In the moderate/severe baseline bloating subgroup, the LSM change from baseline in bloating by week was statistically significant for plecanatide compared with placebo during Weeks 2 through 12 of treatment (except Week 3; Figure 2C).

Plecanatide was well tolerated in the overall population and when patients were stratified by baseline bloating intensity (Table 3). The most frequently reported adverse event in the overall plecanatide‐treated cohort was diarrhea (3.2%), predominantly occurring in the moderate/severe bloating cohort. Only one patient (mild bloating subgroup) withdrew from the study due to diarrhea.

**TABLE 3:** Adverse event summary.

Patient with an AE, *n* (%)	Overall		Mild bloating^a^		Moderate/severe bloating^b^	
	Plecanatide 3 mg (*n* = 313)	Placebo (*n* = 292)	Plecanatide 3 mg (*n* = 94)	Placebo (*n* = 77)	Plecanatide 3 mg (*n* = 219)	Placebo (*n* = 215)
≥ 1 AE	74 (23.6)	49 (16.8)	23 (24.5)	15 (19.5)	51 (23.3)	34 (15.8)
≥ 1 drug‐related AE	13 (4.2)	5 (1.7)	4 (4.3)	1 (1.3)	9 (4.1)	4 (1.9)
≥ 1 serious AE	2 (0.6)	3 (1.0)	1 (1.1)	1 (1.3)	1 (0.5)	2 (0.9)
Discontinuation due to AE	6 (1.9)	2 (0.7)	2 (2.1)	1 (1.3)	4 (1.8)	1 (0.5)
Due to diarrhea AE	1 (0.3)	0	1 (1.1)	0	0	0
Most common AEs^c^						
Diarrhea	10 (3.2)	0	1 (1.1)	0	9 (4.1)	0
Headache	7 (2.2)	3 (1.0)	3 (3.2)	0	4 (1.8)	3 (1.4)
URTI	5 (1.6)	4 (1.4)	3 (3.2)	0	2 (0.9)	4 (1.9)

Abbreviations: AE = adverse event; URTI = upper respiratory tract infection.

^a^ Score, 1–5.

^b^ Score, 6–10.

^c^ ≥ 3.0% of patients in any group.

## 4 Discussion

This study demonstrated that plecanatide was well tolerated and simultaneously and significantly improved abdominal pain, bloating, and CSBM frequency, as indicated using varying thresholds to define response in a population of young adults with IBS‐C and bloating at the start of treatment. The current study affirmed the well‐characterized safety and tolerability profile of plecanatide reported in previous pooled analyses of these two trials, including in patients with bloating [13, 16]. Bloating, one of the most common symptoms reported in patients with IBS‐C [4, 5, 6, 7], negatively affects health‐related quality of life and commonly drives patients to seek medical care [8, 9, 10]. Further, bloating intensity has been shown to correlate with abdominal pain intensity and overall IBS symptom severity (with bloating component excluded) [19]. However, bloating is not part of the strict definition of IBS‐C according to Rome criteria [1]. The current post hoc analysis focused on patients aged 18 to 40 years with IBS‐C with bloating because the condition is more likely to be identified in this population [17, 18].

Guidance from the US Food and Drug Administration (FDA) has recommended that response to pharmacologic treatment in patients with IBS‐C be defined as a composite primary endpoint, with improvement in both abdominal pain (≥ 30% decrease from baseline) and CSBMs/week (increase of ≥ 1/week from baseline) for ≥ 50% of treatment time (e.g., ≥ 6 of 12 weeks) [20]. Composite endpoint assessments in this study included response thresholds exceeding the minimum recommendation for these two symptoms. Bloating was not addressed in the FDA recommendations, but post hoc analyses that incorporate improvements in bloating as part of a two‐symptom composite definition of response have been conducted in IBS‐C studies of lubiprostone (e.g., bloating plus stool frequency) [21] and plecanatide (bloating plus abdominal pain or bloating plus CSBM frequency) [16]. To date, there are only two IBS‐C trials that included bloating as part of a trisymptom endpoint [22, 23]. However, this endpoint was derived from the average of bloating, abdominal pain, and abdominal discomfort scores, whereas bowel movement frequency, a cardinal IBS symptom, was not incorporated [22, 23]. Because bloating is a common, bothersome symptom in patients with IBS‐C, it was included, alongside FDA criteria, in the current study as part of the trisymptom definition of response.

When patients were stratified by baseline bloating intensity, a significantly larger percentage of those treated with plecanatide were trisymptom responders compared with patients treated with placebo. This occurred regardless of baseline bloating intensity for the most stringent definitions assessed (e.g., ≥ 40% improvement from baseline in both pain and bloating scores plus an increase of ≥ 2 CSBMs/week in the same week for ≥ 6 of 12 treatment weeks). Interestingly, in patients with mild bloating, there was no statistically significant difference between plecanatide and placebo for the individual symptom of bloating when assessed by week. This may have been related to a floor effect in the mild bloating subgroup, given that the minimum baseline score for inclusion was 1 and the mean score was 4.3 for both treatment groups. In patients with moderate/severe bloating at baseline, plecanatide simultaneously and significantly improved abdominal pain, bloating, and CSBM frequency versus placebo, using varying thresholds to define response, and improved the individual symptom of bloating when assessed weekly. Overall, the data support that trisymptom responses with plecanatide treatment were maintained, regardless of baseline bloating intensity.

Results of the current study are consistent with a previous analysis of plecanatide in patients with IBS‐C stratified by baseline bloating intensity [16]. In that study, composite responder definitions were restricted to two‐symptom components (i.e., bloating plus abdominal pain or bloating plus CSBM frequency), and inclusion criteria included a broad age range (18–85 years). The current study limited the population to adults ≤ 40 years, given evidence suggesting that bloating intensity decreases with age [17, 24], and it included multiple stringent trisymptom response definitions to better understand the clinical profile of plecanatide in patients with IBS‐C who present with bloating. Although further research is needed, the current study also suggests that guanylate cyclase‐C agonists such as plecanatide may be effective in adults with overlapping DGBIs, such as IBS‐C and functional bloating. A 2025 open‐label trial of linaclotide for overlapping symptoms of functional dyspepsia in patients with IBS‐C supports this concept of treating overlapping DGBI symptoms, including fullness/early satiety and bloating [25].

Limitations of the study include the post hoc nature of the analyses, the lack of health‐related quality of life assessment(s), and a potential floor effect in the mild bloating subgroup. Furthermore, the trisymptom composite endpoint and thresholds evaluated have not been validated. Statistical justification for the included thresholds and power calculations was not conducted, given the exploratory, post hoc nature of the analysis. Although participants were told to maintain a stable dietary intake during the trials, changes in dietary habits could have impacted bloating symptom frequency and intensity. As aforementioned, the current analysis also only focused on adults ≤ 40 years of age. In addition, patients assessed were primarily female and (during the randomized portion of the study) white, similar to previous studies in IBS‐C populations. Future trials should include more heterogeneous populations to validate these findings and assess whether the trisymptom endpoint enhances clinical outcome prediction or offers unique therapeutic insights beyond the standard two‐symptom model.

In summary, bloating is a burdensome symptom experienced by most young adults with IBS‐C. Although not a component of current Rome diagnostic criteria for IBS, this symptom is common and substantially negatively impacts quality of life. Patients should be queried for the presence of bloating, and if present, it should be addressed. For patients with bloating at the start of therapy, this post hoc analysis revealed that plecanatide is efficacious for treating multiple sensory and bowel symptoms, including bloating. Furthermore, improvements were identified across a spectrum of both current FDA guidance and more stringent and rigorous endpoints utilizing a trisymptom score. This trisymptom endpoint enables evaluation of classic and common abdominal and bowel symptoms individually and collectively and should be considered for use in future trials.

## Ethics Statement

Both trials received institutional review board approval and were conducted in accordance with the ethical principles of the Declaration of Helsinki (as amended in 1996).

## Consent

All patients provided written informed consent prior to screening.

## Conflicts of Interest

Darren M. Brenner declares being a consultant and speaker for Salix Pharmaceuticals. Andrea S. Shin declares being a consultant for Ardelyx, has served as an advisory board member for Gemelli Biotech, and has served as an advisor for Medis Labs Inc. Adam P. Laitman is an employee of Salix Pharmaceuticals. Jill K. Deutsch declares having served as an advisory board member for Mindset Health. David C. Kunkel declares serving as a consultant for Ardelyx and Gemelli Biotech and as a speaker for Sanofi, Phathom Pharmaceuticals, and Regeneron Pharmaceuticals, and has received research support from Vanda Pharmaceuticals and Atmo Biosciences.

## Data Availability

Qualified researchers interested in obtaining access to trial data should submit a detailed research proposal and data access request to datasharing@bauschhealth.com. For more information, please see https://www.bauschhealth.com/ESG/access‐to‐clinical‐study‐data/.
